# *Sociedade Portuguesa de Cuidados Intensivos* guidelines for stress ulcer prophylaxis in the intensive care unit

**DOI:** 10.5935/0103-507X.20190002

**Published:** 2019

**Authors:** João João Mendes, Mário Jorge Silva, Luís Silva Miguel, Maria Albertina Gonçalves, Maria João Oliveira, Catarina da Luz Oliveira, João Gouveia

**Affiliations:** 1 Sociedade Portuguesa de Cuidados Intensivos - Lisboa, Portugal.; 2 Departamento de Gastrenterologia, Centro Hospitalar de Lisboa Central E.P.E. - Lisboa, Portugal.; 3 Centro de Medicina Baseada em Evidência, Faculdade de Medicina, Universidade de Lisboa - Lisboa, Portugal.; 4 Departamento de Farmácia, Hospital Prof. Doutor Fernando da Fonseca E.P.E. - Amadora, Portugal.; 5 Associação Portuguesa de Farmacêuticos Hospitalares - Lisboa, Portugal.

**Keywords:** Stress, psychological, Peptic ulcer, Prophylaxis, Intensive care units

## Abstract

Critically ill patients are at risk of developing stress ulcers in the upper digestive tract. Agents that suppress gastric acid are commonly prescribed to reduce the incidence of clinically important stress ulcer-related gastrointestinal bleeding. However, the indiscriminate use of stress ulcer prophylaxis in all patients admitted to the intensive care unit is not warranted and can have potential adverse clinical effects and cost implications. The present guidelines from the *Sociedade Portuguesa de Cuidados Intensivos* summarizes the current evidence and gives six clinical statements and an algorithm aiming to provide a standardized prescribing policy for the use of stress ulcer prophylaxis in the intensive care unit.

## INTRODUCTION

Stress ulcer-related gastrointestinal bleeding is a potential complication of critical illness, for which the pathophysiology is complex. Systemic hemodynamic and local alterations result in gastric mucosal blood flow impairment with subsequent ischemic mucosal injury. However, the crucial factor for the development of ulceration and gastric bleeding is the high gastric intraluminal acidity, which is potentiated by fasting.^(^^1^^)^ This provides the rationale for the use of acid-suppressive drugs for pharmacological prophylaxis.^(^^2^^)^

Endoscopically evident upper gastrointestinal lesions may be found in up to 90% of critically ill patients within 3 days of admission;^(^^3^^)^ less than 50% of patients will have occult bleeding (defined as guaiac-positive gastric aspirate or guaiac-positive stool) and approximately 5%^(^^4,5^^)^ will have overt bleeding (defined as hematemesis, bloody gastric aspirate, melena, or hematochezia). However, this does not necessarily translate into clinically significant gastrointestinal bleeding (deﬁned as overt bleeding in the presence of hypotension, tachycardia or orthostasis, a drop in hemoglobin of > 2g/dL, or the need for surgery),^(^^6^^)^ whose incidence seems to have decreased over the years. In studies published before 1999, the incidence of clinically significant gastrointestinal bleeding was between 2% and 6% in patients not receiving prophylaxis.^(^^6^^)^ However, in studies published since 2001, the incidence has been reported to range between 0.1% and 4% with or without prophylaxis,^(^^7^^)^ which is related to better overall critical care, including the increased use of early enteral feeding. This, along with concerns related to the reported increasing frequency of infectious complications (nosocomial pneumonia and *Clostridium difficile* infections),^(^^8,9^^)^ has challenged the traditional cornerstone of pharmacological prophylaxis with agents that suppress gastric acid for stress ulcer prophylaxis.^(^^10^^)^

This guideline from the *Sociedade Portuguesa de Cuidados Intensivos* aims to summarize current evidence and give clinical recommendations for the use of stress ulcer prophylaxis in the intensive care unit (ICU) to provide a standardized prescribing policy and avoid injudicious use.

## METHODOLOGY

A multidisciplinary task force was assembled. The task force comprised physicians (specialists in gastroenterology and intensive care medicine), nurses, pharmacists and economists with special interest and expertise in stress ulcer prophylaxis and/or evidence-based medicine. All members of the task force declared that no conflict of interest influenced the development of the guidelines.

Task force members participated in a discussion via e-mail, and six clinical questions were built for evidence evaluation. Each working member took charge of one clinical question and built search queries in the PICO (Participants, Interventions, Comparisons, and Outcomes) format.^(^^11^^)^ The availability of a Cochrane review^(^^12^^)^ relevant to the clinical questions was confirmed by searching the Cochrane Database of Systematic Reviews. A further complementary literature search of PubMed^®^ was performed. Trial data identified by the search strategies were considered to represent the best-quality evidence. The Grading of Recommendations Assessment, Development, and Evaluation (GRADE) system principles^(^^13^^)^ was used to assess the quality of evidence from high to very low and to determine the strength of recommendations.

Finally, the task force determined the direction (for or against) and strength (strong or weak) of the recommendations using a two-round (self-administered questionnaire with no meetings among the participants) simple Delphi method.^(^^14^^)^ This was done according to the GRADE system and considered the following factors: evidence quality, certainty in the balance between advantages and disadvantages, certainty or similarity in values and preferences, and resource implications. Arriving at a consensus required an average level of agreement of ≥ 80%. When the agreement level was < 80%, further discussions and voting were conducted.

A strong recommendation was worded as "we recommend" and a weak recommendation as "we suggest".

The key recommendations were presented at the annual symposium of the *Sociedade Portuguesa de Cuidados Intensivos* in Oporto and discussed by the panel and audience members.

## STATEMENTS

### Statement 1

**We recommend** maintaining (or initiating) agents that suppress gastric acid (namely, proton-pump inhibitors) in patients with compelling indications for acid suppression. **Strong recommendation, moderate quality of evidence**.

### Rational

Several clinical situations require gastric acid suppression (namely, proton-pump inhibitors), and indications should be respected, both in the ambulatory and hospital (including intensive care) settings.

Patients with compelling indications include the following:

- Known peptic ulcer disease in the healing phase and maintenance phase in selected circumstances [> 50 years old; multiple comorbidities; persistent symptoms; NSAID-negative and *Helicobacter pylori*-negative ulcers; need to continue NSAID or failure to eradicate *Helicobacter pylori*; ulcers complicated at the outset; and giant (> 2cm), refractory or recurrent ulcers].^(^^15^^)^
- Treatment of *Helicobacter pylori* infection.^(^^16^^)^
- Zollinger-Ellison syndrome and other hypersecretory conditions.^(^^17^^)^
- Gastroesophageal reflux disease and acid-related complications (i.e., erosive esophagitis or peptic stricture)^(^^18^^)^ and Barrett's esophagus.^(^^19^^)^
- Eosinophilic esophagitis.^(^^20^^)^
- Dual antiplatelet therapy or concomitant anticoagulant therapy.^(^^21^^)^

Other approved indications (which should be discussed on a case-by-case basis) include the following:

- Uninvestigated dyspepsia^(^^22^^)^ and epigastric pain syndrome.^(^^23^^)^

Approved indications may vary with specific acid suppressants, and therefore labeling indications should be considered.

### Statement 2

**We recommend** prophylaxis with agents that suppress gastric acid rather than no prophylaxis in patients who have one *major* risk factor or two *minor* risk factors for stress ulceration.

**Strong recommendation, low quality of evidence**.

### Rational

Meta-analysis and systematic reviews^(^^6,24,25^^)^ have consistently shown that agents that suppress gastric acid (namely, histamine-2-receptor antagonists and/or proton-pump inhibitors) are superior to placebos in reducing the risk of clinically significant gastrointestinal bleeding. However, a recent meta-analysis^(^^26^^)^ suggested that in patients receiving enteral feeding, pharmacologic prophylaxis of stress ulcers is not beneficial, and combined interventions may even increase the risk of some infectious complications. This metanalysis has been criticized, and a number of large phase-III trials comparing pharmacological prophylaxis and placebos are under way. Their results and subsequent updated meta-analyses are expected to provide important, more relevant data on the balance between the benefits and harms of stress ulcer prophylaxis.^(^^27^^)^

Importantly, the incidence of stress ulcer-related gastrointestinal bleeding is not equally shared across the spectrum of patients admitted to intensive care, and certain patients appear more at risk for bleeding.

A large multicenter prospective cohort study^(^^4^^)^ identified coagulopathy (defined as a platelet count < 50,000/m^3^, an international normalized ratio greater than 1.5, or a partial thromboplastin time greater than 2 times the control value) and respiratory failure (defined as the need for mechanical ventilation for at least 48 hours) as major risk factors for clinically significant gastrointestinal bleeding. The robustness of these risk factors has been confirmed in at least one additional small observational study.^(^^28^^)^

Older studies have been criticized because clinical practice has undergone major changes^(^^10^^)^ in the last 20 years, which have reduced the incidence of stress ulcer-related gastrointestinal bleeding. Moreover, in line with what was previously described, a recent exploratory randomized clinical trial^(^^29^^)^ comparing pharmacologic prophylaxis (with proton-pump inhibitors) and a placebo in mechanically ventilated critically ill patients anticipated to receive enteral nutrition did not show any benefit (or harm) of acid suppression. Because this was a feasibility trial, no firm evidence could be inferred, and the final conclusion was that it is possible to administer pharmacologic prophylaxis promptly after commencing mechanical ventilation.

Patients with traumatic brain injury (Glasgow Coma Scale score ≤ 8), traumatic spinal cord injury, or burn injury (> 35% of the body surface area) have been routinely excluded from these studies because of a presumed high-risk of stress ulcer-related gastrointestinal bleeding most likely mediated through neurological pathways.^(^^30^^)^ Nevertheless, small randomized controlled trials^(^^31-33^^)^ with different acid suppression regimens have demonstrated significant protection from stress ulcer-related gastrointestinal bleeding in these high-risk populations.

No study has been performed specifically for sepsis; however, stress ulcer prophylaxis has been an integral part of the care of septic patients and is recommended by current guidelines.^(^^34^^)^ This makes sense regarding the new sepsis definitions^(^^35^^)^ in which the infection-related dysregulated host response has to be associated with a severe (life-threatening) organ dysfunction (identified as an acute change in total SOFA score ≥ 2 points), and thus includes multiple risk factors.

The evidence supporting other minor risk factors for stress ulcer-related gastrointestinal bleeding is weak as a result of a high risk of systematic and random errors. However, an increasing number of risk factors is associated with an increased risk of bleeding,^(^^36^^)^ and international guidelines recommended stress ulcer prophylaxis for patients with two or more risk factors.^(^^37^^)^ In the original description of stress-ulcer bleeding, hypotension (alongside sepsis and respiratory failure) was associated with stress-related mucosal damage.^(^^38^^)^ A recent inception cohort study identified the presence of three or more comorbidities (including glucocorticoid therapy), preexisting liver disease, renal failure (with use of renal replacement therapy), and coexisting or acute coagulopathy and higher SOFA-score, as significant risk factors for stress-ulcer bleeding after multivariate analysis.^(^^39^^)^ In another large cohort study,^(^^40^^)^ acute kidney injury (assessed by maximum serum creatinine level) was independently associated with an increased risk of gastrointestinal bleeding in patients mechanically ventilated for more than 48 hours. Additionally, a small prospective randomized trial^(^^33^^)^ demonstrated independent significance for the injury severity score.

### Statement 3

**We recommend** the use of a proton-pump inhibitor when prophylaxis with agents that suppress gastric acid is indicated. **Strong recommendation, low quality of evidence**.

### Rational

The choice of the pharmacological prophylaxis agent should take into account factors related to effectiveness, adverse effects and cost.

Sucralfate, a mucosa-protective agent, alone has traditionally been considered inferior to histamine-2-receptor antagonists for stress ulcer prophylaxis.^(^^6,41^^)^ While this has been challenged in a recent meta-analysis of randomized controlled trials,^(^^42^^)^ the results have been criticized because of significant heterogeneity between studies, of which only three had clinically significant gastrointestinal bleeding as a reported outcome.^(^^43^^)^

The efficacy of proton-pump inhibitors and histamine-2-receptor antagonists in preventing stress-ulcer bleeding in critically ill patients has been compared in several randomized control trials and meta-analyses.^(^^25,44-48^^)^ The most recent and complete meta-analyses of randomized controlled trials^(^^25,44^^)^ consistently demonstrated that proton-pump inhibitors were more effective than histamine-2-receptor antagonists at reducing clinically significant gastrointestinal bleeding, although this was not accompanied by a reduction in ICU mortality or length of stay. The robustness of these conclusions is limited by the trial methodologies, differences between lower and higher quality trials, sparse data and possible publication bias. An ongoing cluster-randomized crossover trial [Australian and New Zealand Intensive Care Society Clinical Trials Group (ANZICS CTG): study number 1415-01] is comparing proton-pump inhibitors and histamine-2-receptor antagonists, and the results are expected to provide more relevant data.^(^^27^^)^

There are multiple pharmacoeconomic analyses^(^^49-51^^)^ focused on the comparison between histamine-2-receptor antagonists and proton pump inhibitors for the prophylaxis of stress ulcerrelated gastrointestinal bleeding. The results are contradictory, mainly due to the use of different clinical inputs, and there is no strong evidence regarding which is the most effective alternative. Data from the most recent meta-analysis of clinical trials indicate that proton pump inhibitors should be used. However, if one relies on a propensity score-matched observational cohort study, histamine-2-receptor antagonists are the preferred option.^(^^51^^)^ The only clear conclusion is that, as the cost of prophylaxis is small when compared to the costs of complications, the most effective alternative will constitute a dominant alternative.^(^^51^^)^

Although the quality of evidence is suboptimal, proton-pump inhibitors have been the preferred regimen in intensive care units across Europe, the United States and Canada.^(^^52,53^^)^ It is acknowledged that the published literature on this issue derives from heterogeneous populations of critically ill patients who may differ from the populations at risk identified by the previous recommendation.

Additionally, the expected adverse effects of proton-pump inhibitors are a concern and must be taken into account. A cohort study^(^^54^^)^ provided evidence of an increase in pneumonia with proton-pump inhibitor use; however, this study was related only to cardiac surgery patients, and confidence intervals were wide. Small randomized trials ^(^^29,55^^)^^)^ and a case-control study showed an increased adjusted risk for *Clostridium difficile* infections during treatment with proton-pump inhibitors, but this was more related to the duration of exposure.^(^^56^^)^

Ultimately, the desirable consequences of stress ulcer prophylaxis with proton-pump inhibitors are expected to outweigh the undesirable consequences among the population at risk.

### Statement 4

**We make no recommendation** regarding specific proton-pump inhibitor regimens.

### Rational

The ideal drug regimen should be effective in reducing the risk of ulceration, with a low potential for adverse effects and drug interactions and pharmacokinetic characteristics that facilitate its use in patients with organ dysfunction; it should also be cost-effective.

There is no direct comparison between different proton-pump inhibitor-based regimens (including drug, dosing, route of administration and galenic formulation), and heterogeneity across studies (comparing proton-pump inhibitors to other regimens) impairs the comparison of effects between the individual proton-pump inhibitor regimens tested to date. An *a priori* defined subgroup analysis of at least one meta-analysis suggests that the route of administration (enteral *versus* intravenous) and dosing (once *versus* twice a day) do not affect the results.^(^^43,45^^)^

In relation to the route of administration, multiple factors (*e.g.,* vasopressor use, altered gastric emptying and motility, feeding tube and nutrient interactions) may influence enteral absorption in critically ill patients, and the intravenous route is generally preferred.^(^^57^^)^ This is disputed by a study showing that, despite a lower bioavailability, enteral lansoprazole suppressed acid in intensive care unit patients better than the intravenous formulation.^(^^58^^)^ However, this has not been confirmed by further studies, and lansoprazole requires a complex and labor-intensive galenic formulation for feeding tube administration.

Due to its safety in (at least moderate) organ dysfunction, lower probability of drug-drug interactions, and available formulations, intravenous pantoprazole (40mg *qd*) may be a reasonable choice.^(^^59^^)^ However, the definitive choice of the specific proton-pump inhibitor regimen should be based on individual patient and medical values, experience, product labeling, cost-benefit analyses, anticipated risks of drug-drug interactions and adverse effects.

### Statement 5

**We suggest** using histamine-2-receptor antagonists in patients with *Clostridium difficile* infection and indications for stress ulcer prophylaxis. **Weak recommendation, very low quality of evidence**.

### Rational

Accumulating evidence suggests that the use of agents that suppress gastric acid may increase the frequency of infectious complications.^(^^8,9,60^^)^ The most recent and comprehensive meta-analysis^(^^61^^)^ found that therapy with agents that suppress gastric acid was associated with a significant risk of *Clostridium difficile* infections but that the risk was lower for histamine-2-receptor antagonists than with proton-pump inhibitors.

In the critically ill population, the increased risk for *Clostridium difficile* infections is still controversial because meta-analysis is weak in detecting a modest increase in these events.^(^^62^^)^ Nevertheless, the risk of *Clostridium difficile* infections remains higher in patients receiving proton-pump inhibitors compared with patients receiving histamine-2-receptor antagonists.^(^^8^^)^ Moreover, observational studies^(^^63,64^^)^ have shown that continued proton-pump inhibitor use during incident *Clostridium difficile* infections increases the risk of recurrence.

Based on available data and given the significant disease burden and mortality associated with *Clostridium difficile* infections, proton-pump inhibitors should be avoided, and histamine-2-receptor antagonists should be the preferred therapy when stress ulcer prophylaxis is indicated.^(^^62^^)^

### Statement 6

**We recommend** stopping prophylaxis with agents that suppress gastric acid when risk factors are no longer present and the patient is receiving enteral nutrition. **Strong recommendation, low quality of evidence**.

### Rational

Acid suppressants are inappropriately continued in a large proportion of patients after the resolution of risk factors and even after intensive care unit or hospital discharge, thus extending the potential risks and costs associated with stress ulcer prophylaxis beyond the intensive care unit.^(^^65^^)^ This is in agreement with studies that have concluded that 88.5% of stress ulcer prophylaxis in nonintensive care unit patients is inappropriate^(^^66^^)^ and that a relatively restrictive stress ulcer prophylaxis program not only reduces inappropriate use without increasing the rates of hospital-related gastrointestinal bleeding but also results in an estimated annualized cost savings of more than US$ 200.000.^(^^67^^)^

As previously described,^(^^26^^)^ there is some evidence to suggest that in patients receiving enteral feeding, pharmacologic stress ulcer prophylaxis is not beneficial, and combined interventions may even increase the risk of some infectious complications. However, the evidence is still insufficient to justify withholding stress ulcer prophylaxis from patients who are at high risk for gastrointestinal bleeding. It is sufficiently compelling to support the cessation of prophylaxis when risk factors are no longer present and the patient is receiving enteral nutrition.

Patients should thus be evaluated daily during multidisciplinary care rounds for the continued need for prophylaxis, and once the patient is receiving enteral nutrition and risk factors are no longer present, stress ulcer prophylaxis should be discontinued. This strategy will reduce the overuse and unnecessary continuation of agents that suppress gastric acid upon discharge and in the outpatient setting.^(^^68^^)^ As one of the more common indications for stress ulcer prophylaxis is mechanical ventilation, extubation is crucial to identify and possibly discontinue acid suppression therapy.^(^^62^^)^

### General algorithm

**Figure 1:**
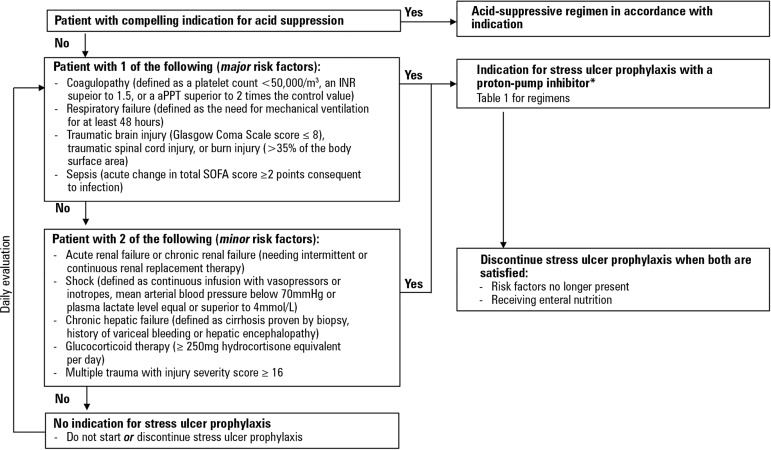
Algorithm for prophylaxis of stress ulcer bleeding in the intensive care unit. * If *Clostridium difficile* infection and indications for stress ulcer prophylaxis favor histamine-2-receptor antagonists. INR - International Normalized Ratio; aPPT - activated partial thromboplastin time; SOFA - Sequential Organ Failure Assessment.

The general algorithm for the prophylaxis of stress ulcer bleeding in the intensive care unit is presented in figure 1. Patients with compelling indications for acid suppression should have an acid-suppressive regimen in accordance with the indication (Statement 1). Then, the risk for bleeding should be considered in each patient; the use of stress ulcer prophylaxis is appropriate for those with high risk. Patients at low risk should not start (or discontinue if previously initiated) stress ulcer prophylaxis (Statement 2). When a stress ulcer prophylaxis is recommended, the use of a proton-pump inhibitor is indicated (Statement 3) with no specific recommended regimen (Statement 4). The exception is cases of *Clostridium difficile* infection, for which histamine-2-receptor antagonists are preferred (Statement 5). Once the patient is receiving enteral nutrition and risk factors are no longer present, stress ulcer prophylaxis should be discontinued (Statement 6). Table 1 compares the different available proton-pump inhibitor- and histamine-2-receptor antagonist-based regimens.

**Table 1:** Comparison of the different available proton-pump inhibitor- and histamine-2-receptor antagonist-based regimens

Drug	Pharmaceutical formulation	Dosing	Dosing and route of administration	Reconstitution and administration	Dose adjustment	Relevant *major* pharmacological interactions (grade 1 - 2 impact)
Pantoprazole	Powder for injection solution	40mg *qd*	Intravenous	Reconstitute 40mg with 10cc of 0.9% NaCl and administer for 2 minutes (if necessary dilute in 100cc of 0.9% NaCl or 5% dextrose in H_2_O)	Hepatic failure (moderate to severe)	Azoles* Reverse protease inhibitors†
	Gastroresistant tablet		Oral*	–		
Omeprazole	Powder for injection solution	40mg *qd*	Intravenous	Reconstitute 40mg with 5cc of 0.9% NaCl and administer for 20 - 30 minutes (if necessary dilute in 100cc of 0.9% NaCl or 5% dextrose in H_2_O)	Hepatic failure (moderate to severe)	Azoles† Reverse protease inhibitors† Clopidogrel‡
	Gastroresistant capsule		Oral	–		
			Endogastric or endojejunal feeding tube	Open capsules, disperse the content in 40mL of non-carbonated water, shake vigorously and allow to stand for 2 minutes (until thick)		
Lansoprozole	Gastroresistant capsule	30mg *qd*	Oral	–	Hepatic failure (moderate to severe)	Azoles† Reverse protease inhibitors†
			Endogastric or endojejunal feeding tube	Open capsules and disperse the content in 40mL of (orange or apple) juice		
	Orodispersible tablet		Oral	–		
			Endogastric or endojejunal feeding tube	Disperse in 10mL of non-carbonated water		
Esomeprazole 40mg i.v. qd	Powder for injection solution	40mg *qd*	Intravenous		Hepatic failure (moderate to severe)	Azoles† Reverse protease inhibitors† Clopidogrel‡
	Gastroresistant capsule		Oral	–		
			Endogastric or endojejunal feeding tube	Open capsules, disperse the granules in 40mL of non-carbonated water		
	Gastroresistant tablet		Oral	–		
Ranitidine	Powder for injection solution	50mg *tid*	Intravenous	Reconstitute 50mg with 20cc of 0.9% NaCl and administer for 5 minutes *Continuous perfusion* : after a 50mg bolus (see above), dilute 150mg to 250cc of 0.9% NaCl or 5% dextrose in H_2_O in perfusion at 10.4cc/hour	Renal failure (clearance < 50mL/min/m^2^)	Azoles†
	Coated tablet	150mg *qd*	Oral	–		
			Endogastric or endojejunal feeding tube	Grind tablets and reduce to powder, and disperse the content in 40mL of non-carbonated water		

* No data on enteral administration; consider alternative drugs;

† consider alternative drugs;

‡ consider substitution by pantoprazole.

NaCl - sodium chroride.

The authors suggest that the practices recommended in this guideline are continuously evaluated and monitored and that this guideline is updated as new evidence becomes available.

## Figures and Tables

**Table:** 

Major risk factor:
- Coagulopathy (defined as a platelet count < 50,000/m^3^, an International Normalized Ratio (INR) greater than 1.5, or a partial thromboplastin time greater than 2 times the control value).
- Respiratory failure (defined as the need for mechanical ventilation for at least 48 hours).
- Traumatic brain injury (Glasgow Coma Scale score ≤8), traumatic spinal cord injury, or burn injury (>35% of the body surface area).
- Sepsis (acute change in total Sequential Organ Failure Assessment - SOFA score ≥ 2 points consequent to infection).
Minor risk factors:
- Acute or chronic renal failure (needing intermittent or continuous renal replacement therapy).
- Shock (defined as continuous infusion with vasopressors or inotropes, mean arterial blood pressure below 70mmHg or plasma lactate level equal to or greater than 4mmol/L).
- Chronic hepatic failure (defined as cirrhosis proven by biopsy, history of variceal bleeding or hepatic encephalopathy).
- Glucocorticoid therapy (≥ 250mg hydrocortisone equivalent per day).
- Multiple trauma with an injury severity score ≥ 16.

## References

[1] Fennerty MB (2002). Pathophysiology of the upper gastrointestinal tract in the
critically ill patient: rationale for the therapeutic benefits of acid
suppression. Crit Care Med.

[2] Cook D, Guyatt G (2018). Prophylaxis against Upper Gastrointestinal Bleeding in
Hospitalized Patients. N Engl J Med.

[3] Eddleston JM, Pearson RC, Holland J, Tooth JA, Vohra A, Doran BH (1994). Prospective endoscopic study of stress erosions and ulcers in
critically ill adult patients treated with either sucralfate or
placebo. Crit Care Med.

[4] Cook DJ, Fuller HD, Guyatt GH, Marshall JC, Leasa D, Hall R (1994). Risk factors for gastrointestinal bleeding in critically ill
patients. Canadian Critical Care Trials Group. N Engl J Med.

[5] Goldin GF, Peura DA (1996). Stress-related mucosal damage. What to do or not to
do. Gastrointest Endosc Clin N Am.

[6] Cook DJ, Reeve BK, Guyatt GH, Heyland DK, Griffith LE, Buckingham L (1996). Stress ulcer prophylaxis in critically ill patients. Resolving
discordant meta-analyses. JAMA.

[7] Faisy C, Guerot E, Diehl JL, Iftimovici E, Fagon JY (2003). Clinically significant gastrointestinal bleeding in critically
ill patients with and without stress-ulcer prophylaxis. Intensive Care Med.

[8] MacLaren R, Reynolds PM, Allen RR (2014). Histamine-2 receptor antagonists vs proton pump inhibitors on
gastrointestinal tract hemorrhage and infectious complications in the
intensive care unit. JAMA Intern Med.

[9] Moayyedi P, Leontiadis GI (2012). The risks of PPI therapy. Nat Rev Gastroenterol Hepatol.

[10] Buendgens L, Tacke F (2017). Do we still need pharmacological stress ulcer prophylaxis at the
ICU. J Thorac Dis.

[11] Guyatt GH, Oxman AD, Kunz R, Atkins D, Brozek J, Vist G GRADE guidelines: 2. Framing the question and deciding on
important outcomes. J Clin Epidemiol.

[12] Toews I, George AT, Peter JV, Kirubakaran R, Fontes LE, Ezekiel JP (2018). Interventions for preventing upper gastrointestinal bleeding in
people admitted to intensive care units. Cochrane Database Syst Rev.

[13] Guyatt GH, Oxman AD, Vist GE, Kunz R, Falck-Ytter Y, Alonso-Coello P, Schünemann HJ, GRADE Working Group (2008). GRADE: an emerging consensus on rating quality of evidence and
strength of recommendations. BMJ.

[14] Boulkedid R, Abdoul H, Loustau M, Sibony O, Alberti C (2011). Using and reporting the Delphi method for selecting healthcare
quality indicators: a systematic review. PLoS One.

[15] Strand DS, Kim D, Peura DA (2017). 25 years of proton pump inhibitors: a comprehensive
review. Gut Liver.

[16] Chey WD, Leontiadis GI, Howden CW, Moss SF (2017). ACG Clinical Guideline: treatment of Helicobacter pylori
infection. Am J Gastroenterol.

[17] Jensen RT, Cadiot G, Brandi ML, de Herder WW, Kaltsas G, Komminoth P, Scoazec JY, Salazar R, Sauvanet A, Kianmanesh R, Barcelona Consensus Conference participants (2012). ENETS Consensus Guidelines for the management of patients with
digestive neuroendocrine neoplasms: functional pancreatic endocrine tumor
syndromes. Neuroendocrinology.

[18] Katz PO, Gerson LB, Vela MF (2013). Guidelines for the diagnosis and management of gastroesophageal
reflux disease. Am J Gastroenterol.

[19] Shaheen NJ, Falk GW, Iyer PG, Gerson LB, American College of Gastroenterology (2016). ACG Clinical Guideline: Diagnosis and Management of Barrett's
Esophagus. Am J Gastroenterol.

[20] Lucendo AJ, Molina-Infante J, Arias Á, von Arnim U, Bredenoord AJ, Bussmann C (2017). Guidelines on eosinophilic esophagitis: evidence-based statements
and recommendations for diagnosis and management in children and
adults. United European Gastroenterol J.

[21] Bhatt DL, Scheiman J, Abraham NS, Antman EM, Chan FK, Furberg CD, Johnson DA, Mahaffey KW, Quigley EM, American College of Cardiology Foundation Task Force on Clinical
ExpertConsensus Documents (2008). ACCF/ACG/AHA 2008 expert consensus document on reducing the
gastrointestinal risks of antiplatelet therapy and NSAID use: a report of
the American College of Cardiology Foundation Task Force on Clinical Expert
Consensus Documents. Circulation.

[22] Talley NJ, Vakil N, Practice Parameters Committee of the American College of
Gastroenterology (2005). Guidelines for the management of dyspepsia. Am J Gastroenterol.

[23] Stanghellini V, Chan FK, Hasler WL, Malagelada JR, Suzuki H, Tack J (2016). Gastroduodenal disorders. Gastroenterology.

[24] Marik PE, Vasu T, Hirani A, Pachinburavan M (2010). Stress ulcer prophylaxis in the new millennium: a systematic
review and meta-analysis. Crit Care Med.

[25] Alhazzani W, Alshamsi F, Belley-Cote E, Heels-Ansdell D, Brignardello-Petersen R, Alquraini M (2018). Efficacy and safety of stress ulcer prophylaxis in critically ill
patients: a network meta-analysis of randomized trials. Intensive Care Med.

[26] Huang HB, Jiang W, Wang CY, Qin HY, Du B (2018). Stress ulcer prophylaxis in intensive care unit patients
receiving enteral nutrition: a systematic review and
meta-analysis. Crit Care.

[27] Marker S, Krag M, Moller MH (2017). What's new with stress ulcer prophylaxis in the
ICU?. Intensive Care Med.

[28] Schuster DP, Rowley H, Feinstein S, McGue MK, Zuckerman GR (1984). Prospective evaluation of the risk of upper gastrointestinal
bleeding after admission to a medical intensive care unit. Am J Med.

[29] Selvanderan SP, Summers MJ, Finnis ME, Plummer MP, Ali Abdelhamid Y, Anderson MB (2016). Pantoprazole or placebo for stress ulcer prophylaxis (POP-UP):
randomized double-blind exploratory study. Crit Care Med.

[30] Schirmer CM, Kornbluth J, Heilman CB, Bhardwaj A (2012). Gastrointestinal prophylaxis in neurocritical
care. Neurocrit Care.

[31] Burgess P, Larson GM, Davidson P, Brown J, Metz CA (1995). Effect of ranitidine on intragastric pH and stress-related upper
gastrointestinal bleeding in patients with severe head
injury. Dig Dis Sci.

[32] Metz CA, Livingston DH, Smith JS, Larson GM, Wilson TH (1993). Impact of multiple risk factors and ranitidine prophylaxis on the
development of stress-related upper gastrointestinal bleeding: a
prospective, multicenter, double-blind, randomized trial. Crit Care Med.

[33] Fabian TC, Boucher BA, Croce MA, Kuhl DA, Janning SW, Coffey BC (1993). Pneumonia and stress ulceration in severely injured patients. A
prospective evaluation of the effects of stress ulcer
prophylaxis. Arch Surg.

[34] Rhodes A, Evans LE, Alhazzani W, Levy MM, Antonelli M, Ferrer R (2017). Surviving Sepsis Campaign: International Guidelines for
Management of Sepsis and Septic Shock: 2016. Crit Care Med.

[35] Singer M, Deutschman CS, Seymour CW, Shankar-Hari M, Annane D, Bauer M (2016). The Third International Consensus Definitions for Sepsis and
Septic Shock (Sepsis-3). JAMA.

[36] Hastings PR, Skillman JJ, Bushnell LS, Silen W (1978). Antacid titration in the prevention of acute gastrointestinal
bleeding: a controlled, randomized trial in 100 critically ill
patients. N Engl J Med.

[37] ASHP Therapeutic Guidelines on Stress Ulcer Prophylaxis (1999). ASHP Commission on Therapeutics and approved by the ASHP Board of
Directors on November 14, 1998. Am J Health Syst Pharm.

[38] Skillman JJ, Bushnell LS, Goldman H, Silen W (1969). Respiratory failure, hypotension, sepsis, and jaundice. A
clinical syndrome associated with lethal hemorrhage from acute stress
ulceration of the stomach. Am J Surg.

[39] Krag M, Perner A, Wetterslev J, Wise MP, Borthwick M, Bendel S, McArthur C, Cook D, Nielsen N, Pelosi P, Keus F, Guttormsen AB, Moller AD, Møller MH, SUP-ICU co-authors (2015). Prevalence and outcome of gastrointestinal bleeding and use of
acid suppressants in acutely ill adult intensive care
patients. Intensive Care Med.

[40] Cook D, Heyland D, Griffith L, Cook R, Marshall J, Pagliarello J (1999). Risk factors for clinically important upper gastrointestinal
bleeding in patients requiring mechanical ventilation. Canadian Critical
Care Trials Group. Crit Care Med.

[41] Cook D, Guyatt G, Marshall J, Leasa D, Fuller H, Hall R (1998). A comparison of sucralfate and ranitidine for the prevention of
upper gastrointestinal bleeding in patients requiring mechanical
ventilation. N Engl J Med.

[42] Huang J, Cao Y, Liao C, Wu L, Gao F (2010). Effect of histamine-2-receptor antagonists versus sucralfate on
stress ulcer prophylaxis in mechanically ventilated patients: a
meta-analysis of 10 randomized controlled trials. Crit Care.

[43] Alhazzani W, Alshahrani M, Moayyedi P, Jaeschke R (2012). Stress ulcer prophylaxis in critically ill patients: review of
the evidence. Pol Arch Med Wewn.

[44] Alshamsi F, Belley-Cote E, Cook D, Almenawer SA, Alqahtani Z, Perri D (2016). Efficacy and safety of proton pump inhibitors for stress ulcer
prophylaxis in critically ill patients: a systematic review and
meta-analysis of randomized trials. Crit Care.

[45] Alhazzani W, Alenezi F, Jaeschke RZ, Moayyedi P, Cook DJ (2013). Proton pump inhibitors versus histamine 2 receptor antagonists
for stress ulcer prophylaxis in critically ill patients: a systematic review
and meta-analysis. Crit Care Med.

[46] Barkun AN, Bardou M, Pham CQ, Martel M (2012). Proton pump inhibitors vs. histamine 2 receptor antagonists for
stress-related mucosal bleeding prophylaxis in critically ill patients: a
meta-analysis. Am J Gastroenterol.

[47] Lin PC, Chang CH, Hsu PI, Tseng PL, Huang YB (2010). The efficacy and safety of proton pump inhibitors vs histamine-2
receptor antagonists for stress ulcer bleeding prophylaxis among critical
care patients: a meta-analysis. Crit Care Med.

[48] Pongprasobchai S, Kridkratoke S, Nopmaneejumruslers C (2009). Proton pump inhibitors for the prevention of stress-related
mucosal disease in critically-ill patients: a meta-analysis. J Med Assoc Thai.

[49] Barkun AN, Adam V, Martel M, Bardou M (2013). Cost-effectiveness analysis: stress ulcer bleeding prophylaxis
with proton pump inhibitors, H2 receptor antagonists. Value Health.

[50] MacLaren R, Campbell J (2014). Cost-effectiveness of histamine receptor-2 antagonist versus
proton pump inhibitor for stress ulcer prophylaxis in critically ill
patients. Crit Care Med.

[51] Hammond DA, Kathe N, Shah A, Martin BC (2017). Cost-effectiveness of histamine2 receptor antagonists versus
proton pump inhibitors for stress ulcer prophylaxis in critically ill
patients. Pharmacotherapy.

[52] Krag M, Perner A, Wetterslev J, Wise MP, Borthwick M, Bendel S, McArthur C, Cook D, Nielsen N, Pelosi P, Keus F, Guttormsen AB, Moller AD, Møller MH, SUP-ICU Collaborators (2015). Stress ulcer prophylaxis in the intensive care unit: an
international survey of 97 units in 11 countries. Acta Anaesthesiol Scand.

[53] Barletta JF, Kanji S, MacLaren R, Lat I, Erstad BL, American-Canadian consortium for Intensive care Drug utilization
I (2014). Pharmacoepidemiology of stress ulcer prophylaxis in the United
States and Canada. J Crit Care.

[54] Bateman BT, Bykov K, Choudhry NK, Schneeweiss S, Gagne JJ, Polinski JM (2013). Type of stress ulcer prophylaxis and risk of nosocomial pneumonia
in cardiac surgical patients: cohort study. BMJ.

[55] Alhazzani W, Guyatt G, Alshahrani M, Deane AM, Marshall JC, Hall R, Muscedere J, English SW, Lauzier F, Thabane L, Arabi YM, Karachi T, Rochwerg B, Finfer S, Daneman N, Alshamsi F, Zytaruk N, Heel-Ansdell D, Cook D, Canadian Critical Care Trials Group (2017). Withholding pantoprazole for stress ulcer prophylaxis in
critically ill patients: a pilot randomized clinical trial and
meta-analysis. Crit Care Med.

[56] Barletta JF, Sclar DA (2014). Proton pump inhibitors increase the risk for hospital-acquired
Clostridium difficile infection in critically ill patients. Crit Care.

[57] Smith BS, Yogaratnam D, Levasseur-Franklin KE, Forni A, Fong J (2012). Introduction to drug pharmacokinetics in the critically ill
patient. Chest.

[58] Olsen KM, Devlin JW (2008). Comparison of the enteral and intravenous lansoprazole
pharmacodynamic responses in critically ill patients. Aliment Pharmacol Ther.

[59] Brett S (2005). Science review: The use of proton pump inhibitors for gastric
acid suppression in critical illness. Crit Care.

[60] Leonard J, Marshall JK, Moayyedi P (2007). Systematic review of the risk of enteric infection in patients
taking acid suppression. Am J Gastroenterol.

[61] Kwok CS, Arthur AK, Anibueze CI, Singh S, Cavallazzi R, Loke YK (2012). Risk of Clostridium difficile infection with acid suppressing
drugs and antibiotics: meta-analysis. Am J Gastroenterol.

[62] Faust AC, Echevarria KL, Attridge RL, Sheperd L, Restrepo MI (2017). Prophylactic acid-suppressive therapy in hospitalized adults:
indications, benefits, and infectious complications. Crit Care Nurse.

[63] McDonald EG, Milligan J, Frenette C, Lee TC (2015). Continuous Proton pump inhibitor therapy and the associated risk
of recurrent Clostridium difficile infection. JAMA Intern Med.

[64] Linsky A, Gupta K, Lawler EV, Fonda JR, Hermos JA (2010). Proton pump inhibitors and risk for recurrent Clostridium
difficile infection. Arch Intern Med.

[65] Farley KJ, Barned KL, Crozier TM (2013). Inappropriate continuation of stress ulcer prophylaxis beyond the
intensive care setting. Crit Care Resusc.

[66] Hong MT, Monye LC, Seifert CF (2015). Acid suppressive therapy for stress ulcer prophylaxis in
noncritically ill patients. Ann Pharmacother.

[67] Buckley MS, Park AS, Anderson CS, Barletta JF, Bikin DS, Gerkin RD (2015). Impact of a clinical pharmacist stress ulcer prophylaxis
management program on inappropriate use in hospitalized
patients. Am J Med.

[68] Heidelbaugh JJ, Inadomi JM (2006). Magnitude and economic impact of inappropriate use of stress
ulcer prophylaxis in non-ICU hospitalized patients. Am J Gastroenterol.

